# Cooperative Extension professionals' knowledge and attitudes toward the opioid epidemic: Implications for capacity development and outreach

**DOI:** 10.3389/fpsyt.2022.958335

**Published:** 2022-08-17

**Authors:** Lisa T. Washburn, Karen L. Franck, Sreedhar Upendram, Jackie N. Yenerall

**Affiliations:** ^1^Department of Family and Consumer Sciences, University of Tennessee Institute of Agriculture, Knoxville, TN, United States; ^2^Department of Agricultural and Resource Economics, University of Tennessee Institute of Agriculture, Knoxville, TN, United States

**Keywords:** opioid epidemic, substance use prevention, opioid crisis, needs assessment, capacity development, Cooperative Extension, Extension educators, professional development

## Abstract

**Introduction:**

Worsening of the opioid epidemic amplifies calls for involvement of the nationwide Cooperative Extension System (Extension) in addressing this crisis. Understanding knowledge and attitudes among Extension professionals who directly interact with communities is critical given identified needs for increased capacity and substantial federal investments supporting Extension's opioid response. This study explored opioid knowledge and attitudes among Extension professionals in one state to identify attitudes and perceptions that may influence community-level efforts.

**Methods:**

An online survey including 25 Likert scale questions about attitudes and beliefs related to substance use was administered to Extension professionals. Questions were categorized into five concept areas: treatment and community support, legal and punitive approaches, substance use as an illness, external causes of substance use, and personal causes of substance use. Descriptive statistics and response frequencies for all variables were calculated. One-way ANOVAs were used to calculate geographic differences between the state's three Extension regions.

**Results:**

Survey responses (*n* = 236) indicated respondents recognized the complexity of the opioid crisis and had favorable attitudes toward treatment and community support approaches. Support for legal and punitive approaches was mixed, as were attitudes toward external and personal causes of substance use. Most indicated needing better resources and more knowledge to engage in work locally.

**Conclusion:**

Increased capacity is needed in Extension to adequately support families and communities dealing with substance use disorder. Findings suggest areas of focus and provide insight for others seeking to develop capacity in opioid response by engaging Extension professionals or other community outreach workers in substance use prevention efforts.

## Introduction

The opioid crisis was declared a national public health emergency in 2017, prompting an infusion of federal funds and empowering federal and state agencies to direct resources in response to the opioid epidemic (1). Despite these efforts, drug overdose deaths continued to worsen during the onset of the COVID-19 pandemic (2). While Americans struggled with pandemic protocols and social isolation, from April 2020 to 2021, drug overdose deaths increased 28.5% nationally while some states saw increases exceeding 50% (3). Tennessee experienced a 33% increase in non-fatal opioid overdoses statewide between March and June of 2020 as compared to the same period in 2019 (4). The worsening opioid epidemic and resulting societal burden amplifies the need for increased efforts of Cooperative Extension and capacity development at the community level to help stem this crisis.

The Cooperative Extension System (Extension) is a nationwide network providing community-based, non-formal education through local Extension offices affiliated with states' land grant universities. Research findings from the university are extended to communities through a partnership between federal, state, and local governments (5). Local Extension efforts take a grassroots approach characterized by county-based Extension educators, in some states referred to as county Extension agents (hereafter, Extension professionals), working in partnership with residents and other stakeholders to increase knowledge, improve practices, and solve problems (6). Agriculture, youth development (4-H), community development, and family and consumer sciences have been the primary focus for Extension education efforts. Campus-based, subject-matter Extension specialists train and support county-level Extension professionals who identify and address community needs through education and outreach typically delivered through direct education and demonstrations (7). With offices in or near most U.S. counties, the footprint of county Extension offices and personnel differs by state (6).

Since 2017, federal funds specifically allocated for the Cooperative Extension System to respond to substance misuse issues primarily flowed through two federal agencies: United States Department of Agriculture (USDA), through the National Institute of Food and Agriculture's Rural Health and Safety Education (RHSE) competitive grants program, and the Substance Abuse and Mental Health Services Administration (SAMHSA) Rural Opioid Technical Assistance (ROTA) grants. Starting in 2017, RHSE began prioritizing funding for projects addressing the opioid epidemic in rural communities (8). In 2018 and 2019, RHSE funding focused exclusively on projects to prevent or reduce opioid misuse (9, 10). From 2017 to 2021 USDA invested $12.6 million in 38 RHSE-funded projects addressing various aspects of substance misuse (11). SAMHSA-funded ROTA grants provided additional resources to states with funded RHSE projects starting in 2018 to “develop and disseminate training and technical assistance for rural communities on addressing opioid issues affecting these communities” (12). Between 2018 and 2020, this funding source infused $22.7 million into the Cooperative Extension System to tackle the opioid crisis across the country.

Extension is recognized as a valued partner in addressing substance use disorder (SUD), with the role of Extension professionals particularly noted (13). Concurrent with the 2017 RHSE shift to prioritize opioid misuse and growing recognition of the long-term impact on communities, the Extension Opioid Crisis Response Workgroup (EOCRW) was formed at the recommendation of the Extension Committee on Organization and Policy. The EOCRW purpose was to “identify and assemble resources to help Extension play a stronger and more strategic role in addressing the opioid crisis, and more generally behavioral health challenges that emerge over time” (“Opioid Response,” paragraph 2). An overwhelming majority of national Extension leaders agreed that Extension should play a role in reducing opioid misuse and overdose in their respective states. However, <24% agreed their Extension system had capacity to respond to the opioid crisis (14). This emphasizes two persisting limitations in Extension's response to the opioid epidemic: (1) lack of knowledge among county-based Extension professionals who typically begin Extension careers without adequate training or professional preparation to address substance misuse at the community level; and (2) the nationwide dearth of Extension faculty and specialists with appropriate expertise to provide much-needed training, support, and educational resources.

Tennessee's experience with the opioid epidemic mirrors many national trends. The overall rate of fatal opioid overdoses increased between 2016 and 2019, but fatal overdoses attributed to prescription opioids declined. During the same period, both the number of prescriptions for opioids and the number of patients filling a prescription for an opioid declined statewide (15). However, the decline in deaths associated with prescription opioids was more than offset by a significant rise in fatal overdoses associated with heroin and fentanyl, which resulted in an overall increase in fatal opioid overdoses. In 2019, Tennessee ranked 13th in the nation for the number of drug overdose deaths, further emphasizing the need to expand Extension's capacity to respond to the epidemic (16).

Understanding knowledge and attitudes among Extension professionals who directly interact with communities is critical given the substantial federal investments supporting Extension's opioid response. Ongoing evolution of the opioid epidemic, with origins in prescription opioids to today's growing use of illicit opioids such as fentanyl, makes Extension's community-level involvement imperative (17).

Gaining a better understanding of current knowledge and attitudes among Extension professionals is an important step in expanding capacity. Therefore, we surveyed county-based Extension professionals in Tennessee to determine knowledge about opioids and the opioid epidemic, identify attitudes and perceptions about opioid misuse and substance use that may support or undermine programs at the community level (i.e., stigma), and explore resource needs of the Extension workforce.

## Methods

### Survey instrument

The study team identified existing survey instruments that measured knowledge, attitudes, and perceptions toward the opioid epidemic and substance use and selected questions based on applicability to county-based Extension professional's roles and community contexts. The final survey included questions from several validated surveys: the Opioid Overdose Knowledge Scale (OOKS) (18), Brief Substance Abuse Attitudes Survey (BSAAS) (19), and Public Views of the Causes of Opioid Pain Reliever Abuse (PVCOPRA) (20). The team developed questions to address emerging issues such as attitudes about justice system involvement and treatment access.

The survey included 25 Likert scale questions about attitudes and beliefs related to substance use (1 = strongly disagree; 5 = strongly agree) categorized into five concept areas: treatment and community support; legal and punitive approaches to address substance use; substance use as an illness; external causes of substance use; and personal causes of substance use (see Table 1). The survey also included two multiple choice questions assessing knowledge, two scenarios with Likert scale responses (1 = definitely not; 5 = definitely) and four scenarios with open-text response (reported elsewhere). Four questions gauged readiness to address the opioid epidemic locally.

**Table 1:** Mean scores and standard deviations for opioid survey questions answered by Extension professionals in a Southern state.

**Question**	**Source of**	**Mean (*SD*)**	**Percentage Agree (A)**
	**Item**		**and Strongly Agree (SA)**
**Treatment and community support**			
The opioid epidemic is a complex problem that needs community support to address locally.	New	4.41 *(0.67)*	45.8% A 48.9% SA
Those arrested for drug-related crimes should be given access to treatment and recovery services while incarcerated.	New	4.20 *(0.74)*	52.5% A 35.4% SA
Medicaid insurance should cover treatment of substance abuse problems, including addiction to prescription pain medications.	New	3.76 *(1.06)*	39.9% A 26.5% SA
Government spending should be increased to improve treatment of substance abuse problems, including addiction to prescription pain medications.	New	3.54 *(1.04)*	39.1% A 16.9% SA
Laws should be passed to protect people from criminal charges for drug crimes if they seek medical help for themselves or others experiencing an overdose.	New	3.13 *(0.94)*	29.4% A 5.9% SA
**Legal and punitive approaches**			
Pregnant women who use opioids or other drugs should be punished.	BSAAS	3.44 *(0.97)*	38.1% A 12.6% SA
Law enforcement does not focus enough on arresting drug dealers who sell prescription pain medication illegally.	PVCOPRA	3.22 *(0.96)*	27.4% A 9.9% SA
A health care provider who has been addicted to opioids should not be allowed to practice again.	BSAAS	2.95 *(0.92)*	22.9% A 4.5% SA
The solution to reducing opioid use is to put drug users in jail.	New	2.46 *(0.99)*	6.7% A 4.5% SA
**Substance use as an illness**			
Alcohol addiction is a treatable illness.	BSAAS	4.12 *(0.77)*	55.1% A 31.1% SA
Opioid addiction is a treatable illness.	BSAAS	3.85 *(0.62)*	70.1% A 9.0% SA
Drug addiction should be treated as a chronic illness, not a crime.	New	3.21 *(1.03)*	32.1% A 9.0% SA
**External causes of substance use**			
Some people do not store prescription pain medication safely in their homes, making these drugs easy for others to take.	PVCOPRA	4.26 *(0.72)*	57.3% A 36.4% SA
Some people do not dispose of prescription pain medication when they are no longer needed, making these drugs easy for others to take.	PVCOPRA	4.12 *(0.70)*	60.4% A 27.0% SA
Health insurance companies are more likely to pay for prescription pain medication than other treatments such as physical therapy or acupuncture.	PVCOPRA	3.76 *(0.96)*	42.7% A 22.7% SA
Pharmaceutical companies do not adequately explain the risks of addiction on labels of prescription pain medications.	PVCOPRA	3.53 *(1.08)*	34.8% A 22.9% SA
Doctors often put patients on prescription pain medication at too high doses.	PVCOPRA	3.45 *(0.83)*	32.1% A 11.3% SA
Doctors often write prescriptions for pain medication without properly examining a patient to assess their need for pain medication.	PVCOPRA	3.44 *(0.97)*	36.2% A 13.4% SA
Pharmaceutical companies promote prescription pain medication without adequate knowledge of their safety and effectiveness.	PVCOPRA	3.31 *(1.05)*	25.3% A 15.6% SA
Doctors do not get enough training about how to prescribe prescription pain medications.	PVCOPRA	3.17 *(0.85)*	25.0% A 6.3% SA
**Personal causes of substance use**			
Some people do not understand how easy it is to become addicted to prescription pain medication.	PVCOPRA	4.31 *(0.62)*	54.1% A 38.7% SA
Some people have a family history that makes them more likely to abuse prescription pain medication.	PVCOPRA	3.89 *(0.91)*	51.8% A 24.1% SA
Some people lack the self-discipline to use pain medication without becoming addicted.	PVCOPRA	3.76 *(0.87)*	48.6% A 18.5% SA
Heroin is so addicting that no one can really recover once he/she becomes an addict.	BSAAS	2.63 *(0.92)*	11.1% A 3.6% SA
Opioid addiction is associated with a weak will.	BSAAS	2.54 *(0.93)*	14.9% A 1.8% SA

### Data collection

Regional directors who serve as overall supervisors for county employees emailed links to the online survey to county-based Extension professionals in Tennessee. The online survey was administered using QuestionPro software. Survey invitations were sent to those on email distribution lists for 4-H, agriculture and natural resources, and family and consumer sciences (FCS) agents and specialists in each of three Extension regions in the state. The study team sent two reminder emails as follow-up, the first 1 week after the initial invitation, and the second a week later. Data were collected in January–February 2021. The study protocol was approved by the University of Tennessee Institutional Review Board (protocol #19-05316-XP).

### Data analysis

Agree and strongly agree responses to survey questions were combined to form a single response category and are reported as favorable responses. Data were analyzed using SPSS. Only answers from completed questionnaires were included in the data analyses. Geographic differences in responses between the state's three Extension regions (western, central, eastern) were calculated using one-way ANOVAs. Differences between educational attainment levels of respondents (Bachelor's degree and master's degree or higher) were calculated using paired *t*-tests.

## Results

Of 258 surveys started, 236 Extension employees completed the surveys (91.5% completion rate). Extension employees who finished the survey included 196 county-based Extension professionals (including county directors); 40 surveys were completed by other Extension employees serving in regional administrative or specialist roles. Employees represented all three regions of the state, were primarily White (91%) and most (70%) had a master's degree or higher. Response rate was estimated at 42.9% based on the combined number of email addresses on the three distribution lists used (*n* = 550). County-based Extension professionals were the primary target group for the survey as they do most of the education and outreach in communities and represent Extension on diverse community groups such as anti-drug coalitions. The response rate for county-based Extension professionals was 70.5% overall (196 responses out of 278 professionals) and 66% for those serving as county directors. There were no significant differences in responses between the three regions of the state.

### Knowledge

Fifteen percent of respondents correctly identified all 8 opioids from a list of 11 drugs. The median number of correct responses was five. Slightly more than half of respondents (51.3%) correctly identified heroin as an opioid. Most respondents identified OxyContin (94.1%), hydrocodone (88.6%) and codeine (73.3%) as opioids. Respondents were also asked to identify the purpose of Narcan or naloxone. Most respondents (80.1%) correctly identified these as opioid overdose reversal drugs.

### Attitudes and beliefs

Questions related to attitudes and beliefs were grouped into five categories: treatment and community support, legal and punitive approaches to address substance use, substance use as an illness, external causes of substance use, and personal causes of substance use. Table 1 contains means, standard deviations, and percent agreement for all survey responses.

#### Treatment and community support

These questions related to attitudes toward substance use disorder treatment and the role of community support. Almost all respondents (94.7%) agreed with the statement that the opioid epidemic is a complex problem that needs community support to address locally. Furthermore, most respondents agreed that people incarcerated for drug-related crimes should have access to treatment and recovery (87.9%). Fewer respondents supported paying for treatment options through expansion of Medicaid (66.4%) and increased government spending (56.0%).

#### Legal and punitive approaches to address substance use

In contrast to treatment and support options, respondents had mixed responses to questions addressing legal and punitive approaches to address substance use. For example, most respondents (88.8%) indicated that incarcerating drug users does not have an impact on reducing opioid abuse, but fewer respondents (35.3%) supported laws to protect people from criminal charges for drug crimes if seeking help for themselves or others experiencing an overdose. Over half of respondents (50.7%) agreed that pregnant women who use opioids or other drugs should be punished, but far fewer (27.4%) supported punitive actions for addicted health care providers like barring them from practicing medicine again.

#### Substance use as an illness

Most respondents believed that alcohol and opioid addiction are treatable illnesses (84.8 and 79.1%, respectively). However, less than half (41.1%) believed that drug addiction should be treated as an illness and not a crime.

#### External causes of substance use

These questions specifically focused on perceived causes of prescription drug misuse and addressed the role of individuals, pharmaceutical companies, healthcare providers, and insurers. Most respondents agreed that people who did not safely store and dispose of prescription opioids in their homes made it easy for others to access these drugs (93.7 and 87.4%, respectively). Respondents were less likely to agree that doctors write prescriptions for opioids at higher dosage than needed (43.4%) or did not adequately assess patients' needs for opioids (49.6%). Respondents were less likely to assign responsibility to pharmaceutical companies; 58.9% agreed that pharmaceutical companies do not properly label the risks of opioids and 40.9% agreed they promote opioids without adequate knowledge about their safety or effectiveness.

#### Personal causes of substance use

These questions related to personal characteristics perceived to contribute to substance use such as lack of knowledge, family history, and lack of willpower. Most respondents (92.8%) agreed that people do not understand how easy it is to become addicted to prescription pain medication. In addition, 75.9% agreed that family history predisposes some people to substance use and 67.1% agreed that some people lack the self-discipline to use pain medication without becoming addicted. In contrast, only 16.7% believed that opioid addiction is associated with a weak will.

### Readiness to address the opioid epidemic

Few respondents indicated they had adequate resources to address the opioid issue in their county. Only 3.8% agreed they had the tools needed to engage in work addressing the opioid epidemic in their community, 9.9% agreed they had adequate knowledge, and 36.9% were comfortable doing work in this area. However, almost half (48.5%) had knowledge of who to partner with at the community-level to address opioid related issues.

Despite the perceived lack of resources, many respondents indicated some level of comfort or willingness to interact with individuals involved in substance misuse or in recovery. For example, almost all (97.7%) responded that they would intervene when encountering youth discussing misusing prescription medications and 72.4% were comfortable talking with someone who was in recovery. In a scenario involving encountering a possible overdose at a training event, more than half (51.8%) said they would be willing to administer Narcan, assuming they had been trained to do so.

Independent sample *t*-tests compared responses between those with a bachelor's degree (*n* = 65) and those with a master's degree or higher (*n* = 165). Significant differences between the two groups were found for two issues—one related to whether Medicaid should cover SUD treatment [*t*_(215)_ = 2.33, *p* < 0.05] and whether opioid addiction is a treatable illness [*t*_(213)_ = 2.06, *p* < 0.05]. For both items, respondents with a bachelor's degree were more likely to report favorable attitudes compared to respondents with a master's degree (mean scores of 4.02 vs. 3.65 for whether Medicaid should cover treatment, and 3.98 vs. 3.79 for whether opioid addiction is a treatable illness).

## Discussion

This is the first published study providing insight to opioid knowledge and attitudes among Extension professionals in Tennessee, and perhaps the first reported within the national Cooperative Extension System to explore knowledge and attitudes among a sample largely comprised of county-based Extension professionals. Increased capacity is needed in Extension to adequately support families and communities struggling to deal with SUD and subsequent outcomes. Findings suggest areas of focus for capacity-building and development efforts.

### Knowledge

Only 10% of survey respondents reported possessing adequate knowledge to address the opioid epidemic. Others have identified gaps in evidence-based practice and make a case for strengthening and expanding Extension capacity for behavioral health (21). These gaps in evidence-based practice are driven, in part, by limited capacity among Extension professionals at the community-level, an issue which must be remedied to accomplish the EOCRW recommendation to embed health promotion and prevention activities throughout Extension (14).

Challenges in preparing professionals to address SUD extend beyond the Extension System. Similar shortfalls have been noted in professional preparation for social workers, nurses, and physicians (22–25). Training can potentially increase knowledge, confidence, and favorable attitudes in working with people with SUD (25–27). Qualified Extension specialists with behavioral health training as well as dedicated resources are critical to support and sustain efforts.

### Attitudes and beliefs

Reducing stigma of SUD is essential to engage communities to address the opioid crisis. Professionals with negative attitudes toward people who use substances are less likely to be motivated to increase their knowledge of substance use problems and apply that knowledge in their work (23). Stigma discourages those with SUD from seeking treatment and limits access to supportive recovery resources (28). Stigma-induced shame harms families impacted by substance use by limiting social interactions and disconnecting them from needed social support and services.

Current public health approaches recognize addiction as a treatable illness, not a crime, with calls for a systems approach addressing social, economic, and environmental determinants of substance use (1). Cooperative Extension's National Framework for Health Equity and Wellbeing confirms this strategy (29). Law enforcement approaches began shifting in 1989 when drug courts changed focus to treatment and recovery instead of punishment (30). In contrast to these shifts in the public health and law enforcement sectors, some survey respondents favored treating addiction as a crime instead of as an illness. However, when survey statements excluded crime and involved specific substances (e.g., alcohol and opioids), more than 79% supported the addiction as illness approach. Enduring stigma toward both justice system involvement and substance use may explain this disparity in responses for similar questions.

Conflated views of SUD and drug-related offenses as moral failings, caused and controllable by the individual, drives stigma (31). Most respondents in a national public opinion survey indicated individuals with SUD bear responsibility for solving it. However, this same group expressed support for various public policy approaches (20). In our study, a minority of Extension professionals (35.3%) supported laws to protect people from criminal charges for drug crimes if seeking help for themselves or others experiencing an overdose, compared to 50.2% from the national public opinion survey (20). This disparity from Extension professionals in the state may reflect a lack of awareness of potential public value of such policies, and limited knowledge of existing state laws, regulations, and good Samaritan policies offering some protection from prosecution (32). This suggests stigma-reducing education needs to extend beyond basic knowledge of opioids and usefulness of naloxone; increased understanding of policy-oriented solutions may be warranted (29). Training that includes empathy-building and case studies that personalize SUD may be an effective method for decreasing punitive attitudes.

### Role of Extension professionals

The opioid crisis has most heavily impacted rural communities; 45% of rural adults and 74% of farmers and farm workers report being directly impacted by opioid abuse through family, friends, or personal struggles with opioid dependence (33). While addressing the complex issue of substance use and prevention may be perceived as primarily the work of Family and Consumer Sciences or 4-H Youth Development Extension professionals, the opioid crisis affects all Extension clientele groups. The EOCRW recommended all Extension staff interacting with the public attain a basic level of behavioral health knowledge and skills, and that health promotion and prevention activities specifically addressing the opioid crisis be embedded within every division of Extension (14). This is a grand challenge; examples of comprehensively equipping Extension professionals with such knowledge and skills tend to be related to broadly applicable program planning activities (e.g., needs assessment and reporting) or policy enforcement (e.g., youth protection). Integrating behavioral health into existing Extension processes or regularly occurring activities is a potential strategy.

Multi-sector partnerships and collaborative capacity building at the community level are necessary to address complex issues like substance use (34). In addition to behavioral health knowledge and skills, Extension professionals may want support for both building and participating in local partnerships with social services, law enforcement, public health, and others. Effectiveness in collaboration is influenced by professional experience and years worked in a community, making retention and turnover highly relevant to plans for training and community-level implementation (35–37).

As evidenced in the EOCRW Final Report, adoption of evidence-based programs and strategies has been limited (14). In addition to planning for capacity building efforts to enhance knowledge and influence attitudes among Extension professionals, states should also consider support for evidence-based programs to reach communities. The tendency toward use of “home-grown” curricula or traditional Extension approaches over evidence-based programs may be influenced by budget for human resources (salary support for program development) but inadequate investment toward licensing and sustaining evidence-based programs. These programs may require additional budget allocations in both the short and long-term and are often supported by limited-duration grant funds. Adoption of evidence-based programs may also be hindered by opinions that evidence-based curricula are unsuitable for Extension contexts due to limited resources (e.g., money, time constraints of Extension professionals) and inflexible fidelity criteria prohibiting adaptation for local audiences (38–41).

Enhancing Extension's response to the opioid epidemic, and substance use in general, likely requires pairing well-designed internally focused educational efforts to increase knowledge and reduce stigma with program materials and approaches developed for implementation within Extension contexts and acceptable to Extension professionals. Educational efforts, materials, and programs should be based on proven efficacy and effectiveness and aligned with national efforts to reduce health inequities (29). Resources are needed to support development of research and practice-based programs, followed by the longer-term process of establishing evidence for effectiveness.

### Limitations

Use of internal email distribution lists and anonymous data collection make us unable to calculate a true response rate. The estimated response rate of 42.9% may be low due to duplicate emails between distribution lists and because Extension agent lists also included specialists and others (e.g., administrators) outside the target group of county Extension agents. Because many Extension professionals in this state are assigned to more than one program area (e.g., family and consumer sciences and 4-H, or agriculture and natural resources and 4-H) we are unable to delineate programmatic differences. The response rate of 66% of county directors and 70.5% for county agents is notable; those in this group may have responsibilities for family and consumer sciences, 4-H and/or agriculture and natural resources and are the main educators for their communities. While most survey questions were extracted from validated scales, the survey was not validated for assessing attitudes and perceptions toward opioid misuse among Extension personnel. Finally, our sample includes unique respondents from one state. Findings may not be generalizable to Extension professionals in other states with different Extension professional demographics. However, given similarities of Extension professionals educational backgrounds and shared organizational values, our findings may prove useful to others seeking to better understand knowledge and attitudes among this group.

## Conclusion

Cooperative Extension is broadly recognized as a valuable community-level partner. Expanding Extension's role in addressing the opioid epidemic requires more than adding substance use or prevention to the lengthy list of issues for which Extension can provide information. Opioid crisis response by Extension personnel can be effective if additional resources and training are commensurate with complexity of the issue and potential public impact to the communities we serve. The opioid epidemic and societal implications are ongoing, and communities served by Extension personnel will likely continue prioritizing substance use prevention. Understanding attitudes and perceptions of those interacting with the public may be an important step in expanding capacity, particularly when the need to address stigma is high. Additional research is needed to understand knowledge and attitudes of Extension professionals across the country to shape the Cooperative Extension system's response to the national opioid crisis.

## Data availability statement

The raw data supporting the conclusions of this article will be made available by the authors, without undue reservation.

## Ethics statement

This study was reviewed and approved by University of Tennessee Institutional Review Board. Written informed consent for participation was not required for this study in accordance with the national legislation and the institutional requirements.

## Author contributions

LW conceptualized the study, led survey development and data collection, and led manuscript preparation. KF contributed to survey development, conducted data analysis, and contributed to manuscript preparation. SU assisted with survey development, data analysis, and contributed to manuscript preparation. JY assisted with survey development and contributed to manuscript preparation. All authors read, contributed, and approved the final manuscript.

## Funding

This work was supported by a Rural Health and Safety Education Grant (No. 2019-46100-30276) from the United States Department of Agriculture National Institute of Food and Agriculture.

## Conflict of interest

The authors declare that the research was conducted in the absence of any commercial or financial relationships that could be construed as a potential conflict of interest.

## Publisher's note

All claims expressed in this article are solely those of the authors and do not necessarily represent those of their affiliated organizations, or those of the publisher, the editors and the reviewers. Any product that may be evaluated in this article, or claim that may be made by its manufacturer, is not guaranteed or endorsed by the publisher.
