# Supplementary material for: Cooperative Extension professionals' knowledge and attitudes toward the opioid epidemic: Implications for capacity development and outreach
Source: Front Psychiatry. 2022 Aug 17;13:958335. doi: 10.3389/fpsyt.2022.958335 (PMC9428283; doi:10.3389/fpsyt.2022.958335)
Supplement: Supplementary file 1 [file Data_Sheet_1.PDF]

Opioid Knowledge & Attitudes Assessment

Which of the following are examples of opioids? (check all that apply)

1. OxyContin
2. Aspirin
3. Hydrocodone
4. Acetaminophen
5. Heroin
6. Vicodin
7. Tramadol
8. Lorcet
9. Codeine
10. Suboxone
11. Naproxen

What is narcan or naloxone used for? (check all that apply)

1. To reverse the effects of an opioid overdose (e.g. heroin, methadone)
2. To reverse the effects of an amphetamine overdose
3. To reverse the effects of a cocaine overdose
4. To reverse the effects of any overdose

Please indicate the extent to which you disagree or agree with the following statements.

	Strongly disagree	Disagree	Neither agree nor disagree	Agree	Strongly agree
Some people do not store prescription pain medication safely in their homes, making these drugs easy for others to take.	❑	❑	❑	❑	❑
Pharmaceutical companies do not adequately explain the risks of addiction on labels of prescription pain medications	❑	❑	❑	❑	❑
Health insurance companies are more likely to pay for prescription pain medication than other treatments such as physical therapy or acupuncture	❑	❑	❑	❑	❑
TennCare (Medicaid) insurance should cover treatment of substance abuse problems, including addiction to prescription pain medications	❑	❑	❑	❑	❑
Heroin is so addicting that no one can really recover once he/she becomes an addict.	❑	❑	❑	❑	❑

Opioid Knowledge & Attitudes Assessment

Please indicate the extent to which you disagree or agree with the following statements.

	Strongly disagree	Disagree	Neither agree nor disagree	Agree	Strongly agree
The opioid epidemic is a complex problem that needs community support to address locally.	❑	❑	❑	❑	❑
Those arrested for drug-related crimes should be given access to treatment and recovery services while incarcerated.	❑	❑	❑	❑	❑
Alcohol addiction is a treatable illness.	❑	❑	❑	❑	❑
Pharmaceutical companies promote prescription pain medication without adequate knowledge of their safety and effectiveness	❑	❑	❑	❑	❑
Government spending should be increased to improve treatment of substance abuse problems, including addiction to prescription pain medications.	❑	❑	❑	❑	❑

Scenario: You are attending a training event in the county you serve. As you are arriving at the location and parking your car, you notice someone in the car next to you who appears to be unconscious. Assuming that you have been trained to use Narcan/Naloxone (the opioid overdose reversal medication), would you be willing to administer the medication if needed?

1. Definitely
2. Probably
3. Unsure
4. Probably Not
5. Definitely Not

Scenario: You are aware of a person in your community who has been in active addiction for a number of years. You have heard that they are now in recovery and they happen to show up at a training event you are sponsoring. Would you be comfortable engaging in conversation with this person about their recovery?

1. Definitely
2. Probably
3. Unsure
4. Probably Not
5. Definitely Not

Opioid Knowledge & Attitudes Assessment

Please indicate the extent to which you disagree or agree with the following statements.

	Strongly disagree	Disagree	Neither agree nor disagree	Agree	Strongly agree
A healthcare provider who has been addicted to opioids should not be allowed to practice again.	❑	❑	❑	❑	❑
Doctors do not get enough training about how to prescribe prescription pain medications.	❑	❑	❑	❑	❑
Doctors often write prescriptions for pain medication without properly examining a patient to assess their need for pain medication.	❑	❑	❑	❑	❑
Some people have a family history that makes them more likely to abuse prescription pain medication.	❑	❑	❑	❑	❑
The solution to reducing opioid use is to put drug users in jail.	❑	❑	❑	❑	❑

Please indicate the extent to which you disagree or agree with the following statements.

	Strongly disagree	Disagree	Neither agree nor disagree	Agree	Strongly agree
Law enforcement does not focus enough on arresting drug dealers who sell prescription pain medication illegally.	❑	❑	❑	❑	❑
Drug addiction should be treated as a chronic illness, not a crime.	❑	❑	❑	❑	❑
Opioid addiction is a treatable illness.	❑	❑	❑	❑	❑
Pregnant women who use opioids or other drugs should be punished.	❑	❑	❑	❑	❑
Some people do not dispose of prescription pain medication when they are no longer needed, making these drugs easy for others to take.	❑	❑	❑	❑	❑

Opioid Knowledge & Attitudes Assessment

You are leading a local youth event. You hear a group of students talking about sharing and misusing prescription pain medications. Would you intervene?

1. Yes
2. No

You answered 'yes' to the previous question. Please share how would you intervene below.*

A high school student with whom you are familiar with shares that their parent is struggling with an opioid addiction. Do you feel that it is within your scope of work to provide resources to this youth?*

1. Yes, definitely
2. Probably
3. Unsure
4. Probably not
5. Definitely not

Please indicate the extent to which you disagree or agree with the following statements.

	Strongly disagree	Disagree	Neither agree nor disagree	Agree	Strongly agree
Some people lack the self-discipline to use pain medication without becoming addicted.	❑	❑	❑	❑	❑
Doctors often put patients on prescription pain medication at too high doses.	❑	❑	❑	❑	❑
Opioid addiction is associated with a weak will.	❑	❑	❑	❑	❑
Some people do not understand how easy it is to become addicted to prescription pain medication.	❑	❑	❑	❑	❑
Laws should be passed to protect people from criminal charges for drug crimes if they seek medical help for themselves or others experiencing an overdose.	❑	❑	❑	❑	❑

Opioid Knowledge & Attitudes Assessment

Scenario: A program volunteer is exhibiting symptoms of impairment at a community event, potentially from substance misuse (e.g. slurred speech, overly drowsy or nodding off, erratic or unusual behavior). What actions would you take after observing these symptoms?*

Scenario: Your county sees a dramatic increase in drug overdoses. The county health council wants to focus on addressing this issue. How might you contribute to these efforts? *

What training have you received to related to the opioid crisis? (Select all that apply)*

1. Narcan/Naloxone
2. Adverse Childhood Experiences (ACEs)
3. Other – In-service training
4. Other – Conference
5. Other Training

Please indicate the extent to which you disagree or agree with the following statements.

	Strongly disagree	Disagree	Neither agree nor disagree	Agree	Strongly agree
I have the tools I need to engage in work to address opioids in my community.	❑	❑	❑	❑	❑
I am uncomfortable doing work to address the opioid issue.	❑	❑	❑	❑	❑
I know enough about opioids to effectively engage with my community to address the issue.	❑	❑	❑	❑	❑
I know who to partner with in my community to address opioid-related issues.	❑	❑	❑	❑	❑

*Responses to these questions are not reported the article.
